# Supplementary figures and images for: Oxygen-binding proteins aid oxygen diffusion to enhance fitness of a yeast model of multicellularity
Source: PLoS Biol. 2025 Jan 30;23(1):e3002975. doi: 10.1371/journal.pbio.3002975 (PMC11781632; doi:10.1371/journal.pbio.3002975)

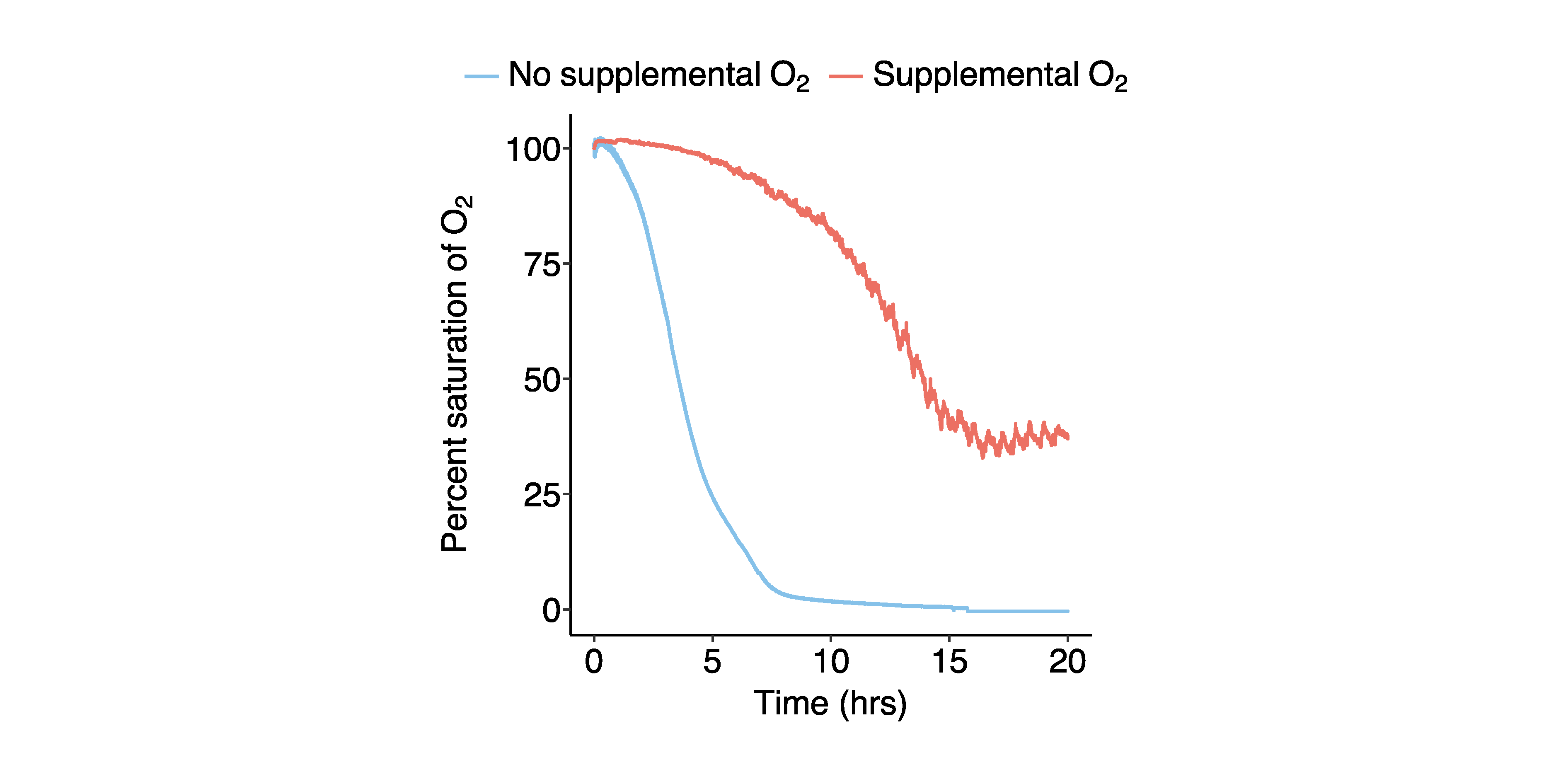

Supplement: S1 Fig — Without supplemental oxygen, the majority of a growth cycle is spent at oxygen levels under <5% saturation (<0.0125 mM). With supplemental oxygen, the majority of a growth cycle is spent at greater than 50% oxygen saturation (>0.125 mM) and average oxygen levels do not drop below 32% saturation (0.08 mM). Data taken from Bozdag and colleagues [17]. Five replicate measurements of oxygen profiles taken with fiber-optic optodes were averaged to produce each oxygen profile. The data underlying this figure can be found in S1 Data and at http://zenodo.org/records/14512540. (TIFF) [file pbio.3002975.s003.tiff]

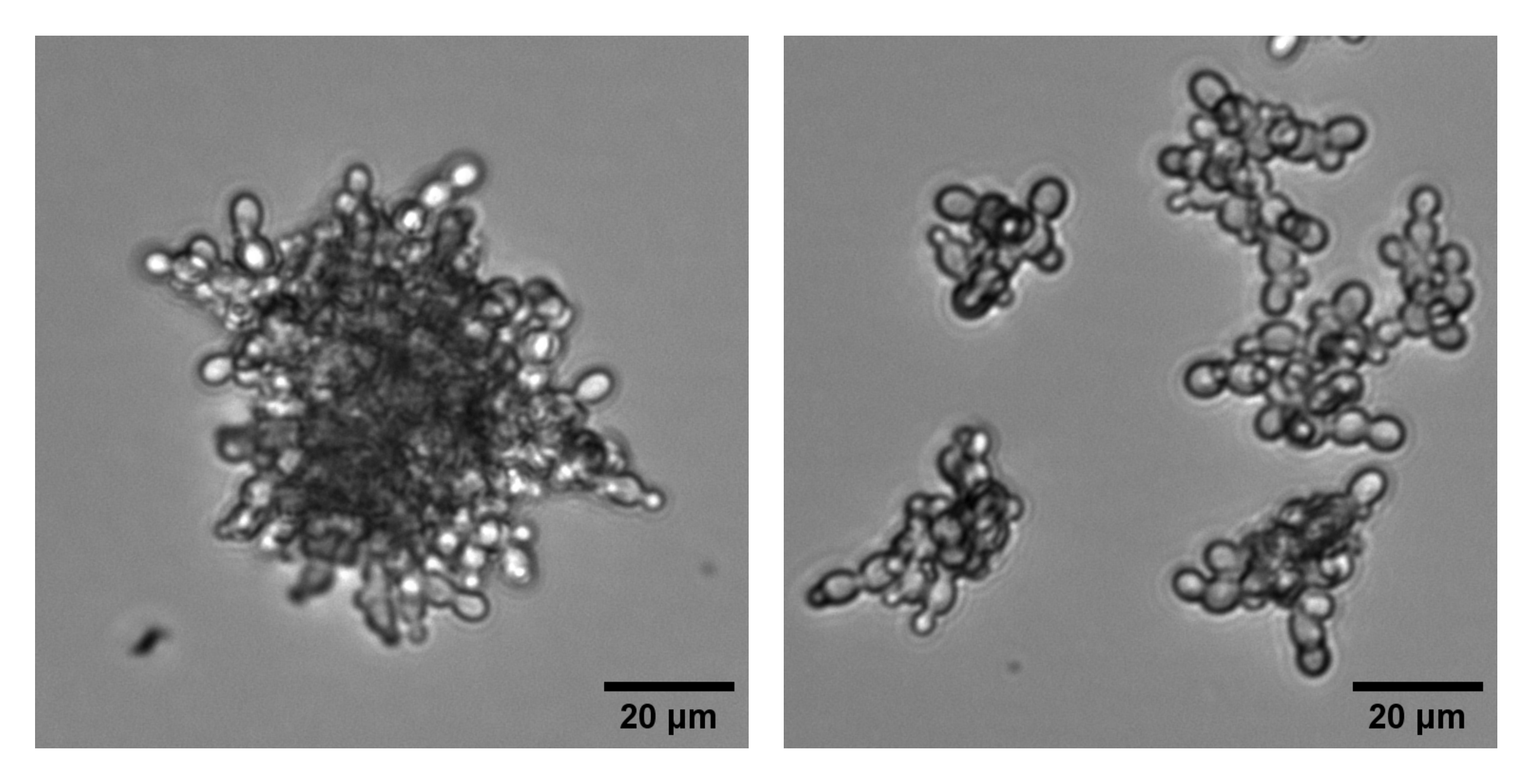

Supplement: S2 Fig — Deletion of the BUD8 gene, which encodes a protein that plays a role in pole selection for budding, results in smaller snowflake yeast clusters. Deletion results in daughter cells back-budding towards mother cells thus fracturing clusters and creating a smaller cluster phenotype. (TIFF) [file pbio.3002975.s004.tiff]

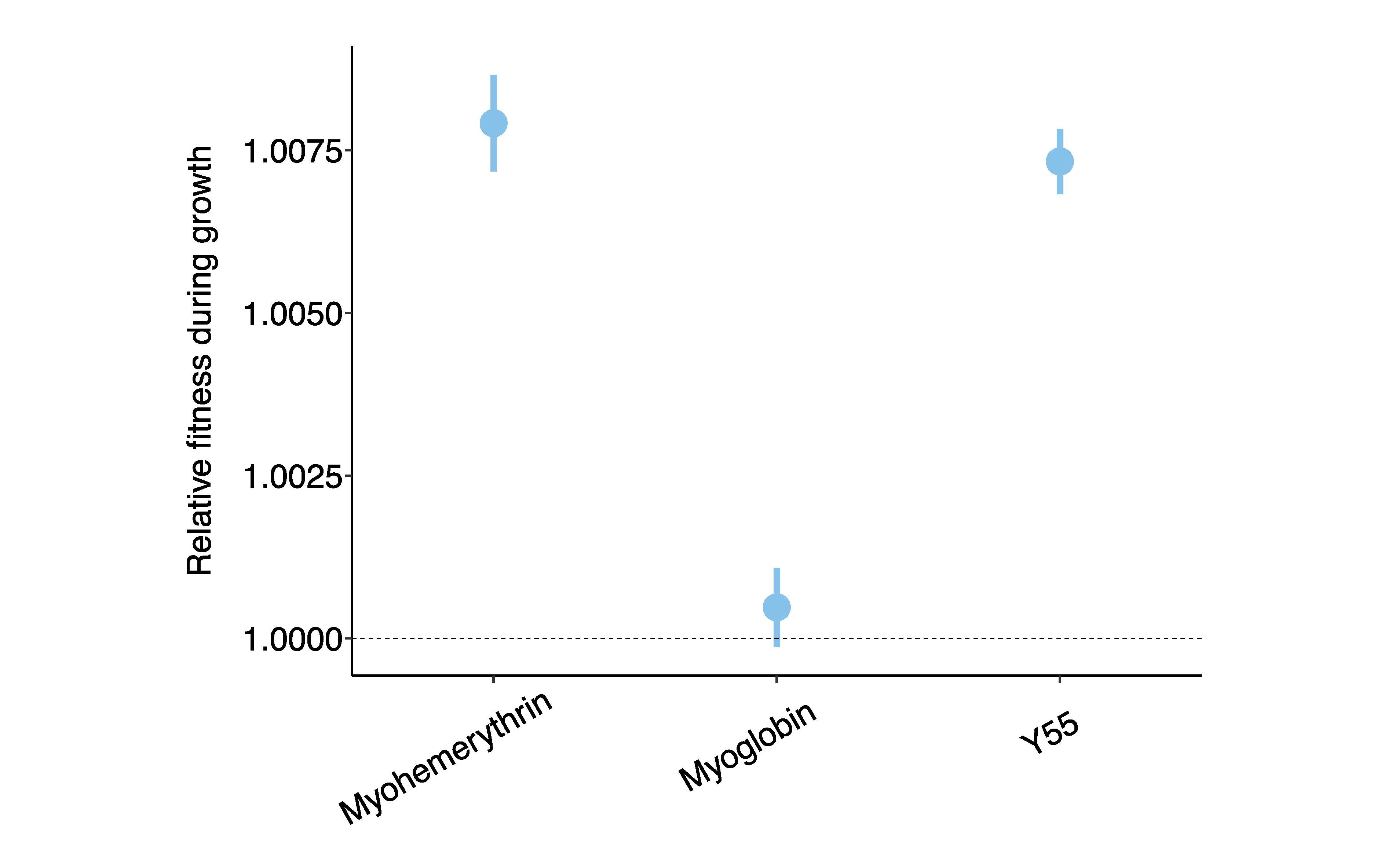

Supplement: S3 Fig — The fitness of unicellular strains when grown in YEP-Dextrose when oxygen was not required. Y55 base strain unicells and myohemerythrin-bearing unicells showed a similar fitness advantage of approximately 0.7% over GFP-bearing competitors, indicating a low cost to myohemerythrin, while the cost of myoglobin expression is roughly equivalent to GFP (one-sample t tests, p = 0.000018 t = 23.89, p = 0.16 t = 1.75, and p = 0.0000052 t = 32.7 for myohemerythrin, myoglobin, and Y55, respectively). This cost of myoglobin is significantly lower than was observed when grown in YEP-Glycerol under low oxygen conditions, while the cost of myohemerythrin is similar. The data underlying this figure can be found in S1 Data and at http://zenodo.org/records/14512540. (TIFF) [file pbio.3002975.s005.tiff]

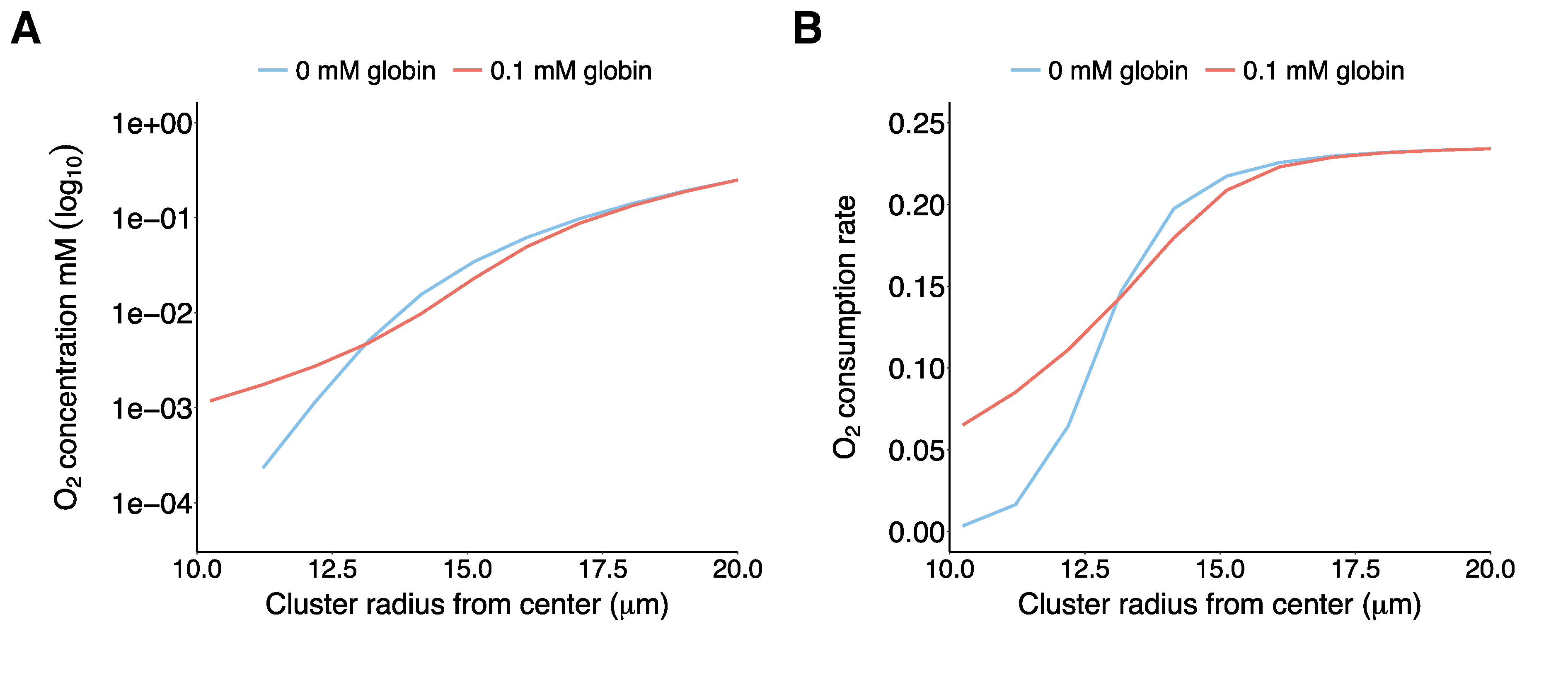

Supplement: S4 Fig — (A) The expression of globin steepens the oxygen gradient at the surface of a cluster, while shallowing the oxygen gradient deeper within a cluster. As the Monod constant of yeast oxygen consumption corresponds to a low oxygen concentration of approximately 3*10−3 mM, the oxygen consumption rate (B) of the deeper portions of the cluster with low oxygen is affected much more strongly than the consumption rate of the shallower portions with high oxygen. Thus, the increase in oxygen consumption deep within the cluster outweighs the slight decrease in oxygen consumption close to the surface of the cluster. The data underlying this figure can be found in S1 Data and at http://zenodo.org/records/14512540. (TIFF) [file pbio.3002975.s006.tiff]
