# Supplementary material for: Oxygen-binding proteins aid oxygen diffusion to enhance fitness of a yeast model of multicellularity
Source: PLoS Biol. 2025 Jan 30;23(1):e3002975. doi: 10.1371/journal.pbio.3002975 (PMC11781632; doi:10.1371/journal.pbio.3002975)
Supplement: Attachment — Submitted filename: Wong et al. Review.pdf [file pbio.3002975.s007.pdf]

**Summary:**

The report by *Whitney Wong et al.* focuses on a relevant theme of evolutionary developmental biology: How oxygen permeability limited/facilitated the evolution of large and likely complex multicellular organisms. The collected experimental and theoretical evidence presented in the report led to a fascinating hypothesis on oxygen-binding proteins' role during this evolutionary transition. Authors found that the exogenous expression of oxygen-binding proteins gave a higher fitness advantage to large multicellular groups at high $O_2$ levels than at low $O_2$ levels. This was not the case for small multicellular groups since the expression of oxygen-binding proteins did not provide any significant fitness advantage under any circumstances. Exploring more in-depth the potential dynamics of oxygen diffusion in the presence of heme-proteins using biophysical modelling, the authors argue that oxygen-binding protein played a crucial role in the evolution of complex multicellularity during the Ediacaran period once oxygen levels had significantly increased.

All the following comments and points are meant to increase the quality of the already exciting work I had a chance to read. I enjoyed reading and studying for this report, and hopefully, I have accomplished my task by providing a helpful review.

**Specific comments:**

The synthetic approach used in this work is fascinating and engaging, although explaining how the specific oxygen-binding proteins were chosen could be helpful. Still, I appreciated using two different proteins from different organisms instead of one only. On a side note, I found it interesting that myoglobin and myohemerythrin showed similar results in the experimental work.

- **Minor point:** Assuming that myohemerythrin has different kinetic properties than myoglobin, would it be possible to run the model for myoH? Given the experimental evidence, it should align with the trends of myoG despite potential biochemical differences. Would you expect differences? If yes, could these inform us on the evolution of oxygen-binding proteins?

As pointed out in the manuscript, oxygen diffusion can be limited by *size* increase. However, in the context of the report's question, '*size*' can be applied to spherical multicellular groups (the valid approach used in the biophysical model), where *size* directly reflects *cell layers* or *thickness* (e.g., Lines 312-314). This distinction made me observe the microscopy images shown in Fig 1C closely and return to those previously published in *Bozdag et al. 2021*. The wild type suggests a strong association between morphology and permeability, such that those cells that are close (i.e., below only one cell) or directly exposed to the environment can show/or not show signs of respiration. In *Bozdag et al. 2021*, this is the case for intermediate $O_2$ conditions. How can morphological evolution potentially limit the adverse effects of poor oxygenation on multicellular growth? Can limited accessibility to oxygen constrain the evolution of specific morphs, such that growing as a *spherical* body would have been negatively selected in a low-oxygen environment? This seems to be a relevant aspect that might need to be expanded if space allows.

**Relevant to Fig1:**

- **Minor point:** It would be helpful to show representative micrographs of MitoLoc yeast expressing Myoglobin and all genotypes under high $O_2$ levels. If space is a limitation, a supplementary panel could be provided.
- **Minor point:** The figure caption of Fig. 1A needs to include information reported in the main text. I don't see the point of having such a level of detail in a main figure without making it directly understandable within the figure caption. The figure could be simplified, and the additional information could be moved to a supplementary figure.

- **Minor point:** Currently, the figure caption indicates that Fig 1B reports mean with SD. However, I interpreted these to be boxplots reporting IQR, medians, and whiskers (?). Moreover, I couldn't find the number of biological replicates. Are individual points considered biological replicates? This would need to be clarified.
- **Minor point:** It could be convenient to consider making images accessible to colourblind readers. The reported micrographs will convey little if no message at all. I list the common potential colorblind-friendly two-colour alternatives: Green/Magenta, Yellow/Blue, and Red/Cyan. Fig 3 has a similar issue but is less limiting than in Fig 1C. I am sure you already know, but in ImageJ, you can recolour individual channels easily (Image>Color>Channel Tools).

Fig. 2B reports the exciting contrast relative to the fitness gains between low and supplemental $O_2$ in normal and small snowflakes. However, I found it difficult to understand why this result was unexpected, even considering the cited references (Lines 156-158).

Normal heme-expressing snowflake yeast gains a fitness advantage under low $O_2$ levels relative to the wild type (Lines 138-142). Moreover, the significant difference in permeability shown in Figs 1B and C is detected under low $O_2$ in contrast to the supplemental $O_2$ level condition, which shows no significant difference.

**Main-minor point:** I consider the $O_2$ levels the primer limiting factor for its diffusion, even if myoH or myoG are abundant. This means that $O_2$ needs to be available to these proteins to observe any effect. In low oxygen conditions, $O_2$ is likely enough only to sustain the physiological activity of cells sitting at the multicellular group periphery.

The results obtained from the model go in this direction (Fig. 3B). The presence/absence of heme proteins is relevant if enough oxygen can be transported. This is also congruent with the observation that, in small snowflakes, there is (yet a non-significant) increase in fitness at low $O_2$ for myoglobin.

Importantly, my point is still congruent with the authors' conclusion, which I find valid and interesting; I am only raising the question of whether the results from the biophysical model could already be expected by considering the experimental data and previous literature. My specific knowledge of the evolution of multicellularity and the constraints imposed by oxygen availability is likely less than that of the authors; thus, I could be missing some additional information to properly follow the above-mentioned transition.

**Additional minor points:**

**Fig. 3** – Expressing '*cluster radius from the centre*' as negative values seems odd (i.e., reporting distances using negative values). Since all graphs are symmetric to 0, they could be halved (as in Fig S3).

Line 157 – ***Intermediate*** is likely ***low*** in this context.

**Methods** – It could be relevant to cite or describe how distances were measured in Fig. 1B.
