# Supplementary material for: Oxygen-binding proteins aid oxygen diffusion to enhance fitness of a yeast model of multicellularity
Source: PLoS Biol. 2025 Jan 30;23(1):e3002975. doi: 10.1371/journal.pbio.3002975 (PMC11781632; doi:10.1371/journal.pbio.3002975)
Supplement: Attachment — Submitted filename: Responseletter_2024-10-30_Final.docx [file pbio.3002975.s008.docx]

We thank the reviewers for their detailed responses, and the journal and editor for their suggestions regarding additional experiments. We have performed a number of additional experiments to address the cost of myoglobin, myohemerythrin, and GFP expression, the benefits these proteins present in unicellular context, and dose-dependence of the effect of their expression. Together with a number of edits regarding clarity and interpretation, we believe this greatly strengthens the manuscript.
 **General reviewer and editor comments:**

First, we clarified why our results are surprising / paradoxical. Specifically, it is counterintuitive that proteins which enhance oxygen diffusion and metabolic availability would provide their greatest benefit when oxygen is the most abundant, and thus most capable of diffusion without the help of such proteins, as opposed to when it is scarce and more limited by diffusion. We explain that this counterintuitive result reflects their primary role in alleviating *anatomical* diffusion constraints rather than *environmental* oxygen limitations. Even when environmental oxygen is abundant, diffusion through tissues can still limit its availability to internal cells, making enhanced diffusion particularly valuable when oxygen is abundant. We have clarified this throughout the paper- hopefully you agree with us that this result is indeed surprising, at least before it is explained (we honestly expected to find the opposite result when we started these experiments, so we truly were surprised by this!).

In response to reviewer and editor comments, we performed two major new experiments. First, we examined protein expression costs by competing unicellular yeast expressing myoglobin or myohemerythrin against GFP-bearing controls under different metabolic conditions. We tested these strains in YEP-Glycerol (where cells must respire) under both low and supplemental oxygen conditions, and in YEP-Dextrose (where cells primarily ferment glucose, making respiratory capacity less important). This approach allowed us to separate the metabolic effects of oxygen-binding proteins from their basic expression costs. Second, we investigated dose-dependent effects by placing myoglobin under the control of a β-estradiol inducible promoter in full-sized snowflake yeast. While moderate levels of myoglobin expression increased fitness at high oxygen levels, excessive expression became detrimental. This result both reinforces the benefit of myoglobin expression for multicellular yeast and reveals a limitation of our model system - very high expression levels may create its own costs, possibly by depleting available heme, reducing the pool available for other essential respiratory proteins.

We have significantly enhanced our methodology descriptions throughout the manuscript. In particular, we have:

1. Provided detailed protocols for oxygen measurement and diffusion depth calculations
2. Clarified our strain construction and genetic modification procedures
3. Added new explanations of our competition assays and fitness calculations
4. Included explicit parameter values and equations used in our mathematical modeling
5. Enhanced figure captions to better describe experimental procedures

In addition to these major experimental and methodological additions, we have made numerous other improvements throughout the manuscript in response to reviewer feedback. We have expanded our introduction to better contextualize the evolution of oxygen-binding proteins, improved our discussion of Proterozoic environmental conditions, clarified our statistical analyses, enhanced our figure presentation including colorblind-friendly modifications, and refined our interpretations of the experimental results. We have also restructured the manuscript as a ‘Short Report’ to provide a more focused presentation of our key findings regarding the relationship between oxygen-binding proteins, multicellularity, and environmental oxygen levels.

Given the focused scope of this revised, shorter format paper, we concentrated our additional experiments on establishing the relationship between oxygen-binding proteins and multicellular size through unicellular controls and dose-dependent expression studies. While several interesting additional experiments were suggested by the reviewers, such as exploring alternative methods of size modification and expanding our parameter space exploration for myohemerythrin modeling, we determined these would be better suited for future work that can more thoroughly examine these aspects.

Thank you all again for your thoughtful suggestions, they have significantly improved our paper! For our specific comments, referee comments are in **black**, our comments are in **blue**, and quotes from the paper are in **purple**.

**Guidance from the Academic Editor**

Thanks, this was very helpful. Below we explain how we addressed each AE suggestion.

 a) The AE said: I find the following statement (and repeated several times in the text) a bit annoying: “Experiments show that, paradoxically, oxygen-binding proteins confer a greater fitness benefit for larger organisms under high, not low, O2 conditions.” To me, this is completely expected, for the same reasons that their model suggests. I suggest deleting "paradoxical" to make it less confusing.

We appreciate your insight regarding our presentation of these results as "paradoxical." We have significantly expanded our explanation of why the benefits of oxygen-binding proteins under high oxygen conditions initially seemed counterintuitive - why would proteins that enhance oxygen diffusion provide their greatest benefit when oxygen is most abundant, as opposed to when it is most diffusionally limiting? We now better explain how this apparent paradox is resolved by considering anatomical versus environmental oxygen limitations, helping readers understand both our initial reasoning and the broader implications of our findings.

b) "re-run the model using myohemerythrin" (rev #1) - AE thinks this is optional.

While re-running our simulations with myohemerythrin would be interesting, we decided against this expansion given the focused scope of this short report. The kinetic and diffusional parameters for myohemerythrin are poorly constrained compared to the very well-studied vertebrate myoglobin, and systematically exploring this large parameter space (three free parameters alongside our existing three variables of protein concentration, oxygen level, and radius) would be better suited for future work.

c) The AE said "With regard to reviewer 3, I also wondered about the effects of the O2-binding proteins on the growth dynamics of both snowflake and ancestral yeast." It would be good to assess this.

We have conducted new experiments examining protein expression costs by competing unicellular yeast expressing myoglobin or myohemerythrin against GFP-bearing controls under different metabolic conditions. These experiments helped separate the metabolic effects of oxygen-binding proteins from their basic expression costs while validating our key findings.

d) "robust measurement of O2 diffusion depth" (rev #3) - AE thinks this is optional.

We have significantly enhanced our description of oxygen diffusion depth measurements in the methods section, providing a detailed explanation of how depths were calculated from cluster imaging and analysis, but did not conduct additional experiments.

e) "try a non-bud8 snowflake strain" (rev #3) - AE thinks this is optional.

Rather than constructing additional small-cluster mutants, we addressed potential bud8-specific effects through our new unicellular strain experiments. These serve as an even more stringent test by completely eliminating any confounding effects of multicellular organization while still allowing us to test baseline costs and benefits of oxygen-binding protein expression.

f) "use an inducible promoter to titrate the O2-binding proteins more subtly" (rev #3) - the AE says "this would be a nice ending to the paper. Their model predicted dosage dependence, and if that is demonstrated experimentally, it will make the paper stronger."

We have implemented this excellent suggestion about testing dose-dependent effects by placing myoglobin under control of a β-estradiol inducible promoter. While the shift from low to moderate levels of myoglobin expression indeed increased fitness at high oxygen levels, very high expression became detrimental. This result both reinforces the dose-dependent benefit of myoglobin expression for multicellular yeast within the low to moderate end of the expression range, and reveals a limitation of our model system - very high expression levels may be costly. We hypothesize that it depletes available heme, reducing the pool available for other essential respiratory proteins and thus reducing fitness.

**Detailed response to referee comments**

Reviewer #1:

Summary:
The report by Whitney Wong et al. focuses on a relevant theme of evolutionary developmental biology: How oxygen permeability limited/facilitated the evolution of large and likely complex multicellular organisms. The collected experimental and theoretical evidence presented in the report led to a fascinating hypothesis on oxygen-binding proteins' role during this evolutionary transition. Authors found that the exogenous expression of oxygen-binding proteins gave a higher fitness advantage to large multicellular groups at high O2 levels than at low O2 levels. This was not the case for small multicellular groups since the expression of oxygen-binding proteins did not provide any significant fitness advantage under any circumstances. Exploring more in-depth the potential dynamics of oxygen diffusion in the presence of heme-proteins using biophysical modelling, the authors argue that oxygen-binding protein played a crucial role in the evolution of complex multicellularity during the Ediacaran period once oxygen levels had significantly increased.

All the following comments and points are meant to increase the quality of the already exciting work I had a chance to read. I enjoyed reading and studying for this report, and hopefully, I have accomplished my task by providing a helpful review.

Thank you for your thoughtful and encouraging summary of our work. Your constructive feedback has helped us strengthen both the presentation and substance of our findings. As detailed below, we've carefully addressed each of your suggestions. In cases where we were unable to include certain experiments, please know that we considered them carefully but determined they would be better suited for future work given the focused scope of this brief report.

Specific comments:

The synthetic approach used in this work is fascinating and engaging, although explaining how the specific oxygen-binding proteins were chosen could be helpful. Still, I appreciated using two different proteins from different organisms instead of one only. On a side note, I found it interesting that myoglobin and myohemerythrin showed similar results in the experimental work.

• Minor point: Assuming that myohemerythrin has different kinetic properties than myoglobin, would it be possible to run the model for myoH? Given the experimental evidence, it should align with the trends of myoG despite potential biochemical differences. Would you expect differences? If yes, could these inform us on the evolution of oxygen-binding proteins?

That’s a cool idea, and one we debated doing prior to our initial submission. While it would be possible to perform simulations using varied kinetic parameters, myohemerythrin is not as well-characterized as myoglobin. In particular, our model requires diffusion constants which must be directly measured in the dense milieu of the cell full of potential binding partners, and separate empirically measured association and dissociation rate constants. This data does not exist in sufficient detail, so we would have to perform large sweeps of parameter space with six dimensions rather than three if we were to run these simulations. We believe the large scale of this potential parameter space, and the fundamental uncertainty of where myoglobin would land in it, leaves its exploration beyond the scope of the current paper but of interest for future work.

As pointed out in the manuscript, oxygen diffusion can be limited by size increase. However, in the context of the report’s question, ‘size’ can be applied to spherical multicellular groups (the valid approach used in the biophysical model), where size directly reflects cell layers or thickness (e.g., Lines 312-314). This distinction made me observe the microscopy images shown in Fig 1C closely and return to those previously published in Bozdag et al. 2021. The wild type suggests a strong association between morphology and permeability, such that those cells that are close (i.e., below only one cell) or directly exposed to the environment can show/or not show signs of respiration. In Bozdag et al. 2021, this is the case for intermediate O2 conditions. How can morphological evolution potentially limit the adverse effects of poor oxygenation on multicellular growth? Can limited accessibility to oxygen constrain the evolution of specific morphs, such that growing as a spherical body would have been negatively selected in a low-oxygen environment? This seems to be a relevant aspect that might need to be expanded if space allows.

We appreciate your insightful questions about morphology and oxygen diffusion. While the microscopy images in both our current work and Bozdag et al., 2021 show flattened clusters (which are much easier to image!), living snowflake yeast clusters have a much more open structure, with approximately 70% free space (Bozdag et al., 2023). This means that oxygen accessibility in living clusters is likely better than what might be inferred from our flattened microscopy images, though the overall pattern of peripheral versus interior cell respiration remains informative.

You raise an interesting point about how morphology could evolve in response to oxygen availability. While different geometries (planar, cylindrical, or branching) might indeed provide varying degrees of oxygen access, our current work focuses specifically on the role of oxygen-binding proteins in enhancing diffusion within a given geometry. We chose to model spherical geometry as it best matches our experimental system and provides a conservative estimate of diffusion limitation. Future work expanding our model to different geometries could provide valuable insights into how morphological evolution might interact with facilitated oxygen diffusion to influence multicellular evolution, but would probably be beyond the scope of the current ‘Short Report’ style paper.

Relevant to Fig1:

• Minor point: It would be helpful to show representative micrographs of MitoLoc yeast expressing Myoglobin and all genotypes under high O2 levels. If space is a limitation, a supplementary panel could be provided.

We appreciate this suggestion, but have chosen not to include additional micrographs for an important methodological reason. The natural variation in size, shape, and fluorescence intensity between individual clusters is typically larger than the subtle differences in oxygen diffusion depth across genotypes under high oxygen conditions. This means that selecting "representative" images could inadvertently cherry-pick examples that suggest visual differences that don't exist in the aggregate data. Instead, we've chosen to show images only where the differences are large enough to be clearly visible by eye, as in our current comparison between wild-type and oxygen-binding protein expressing clusters under low oxygen. This approach aligns with our quantitative data in Figure 1B (see below), which shows that while statistically significant differences emerge when analyzing many clusters, individual clusters often overlap substantially in their characteristics.

• Minor point: The figure caption of Fig. 1A needs to include information reported in the main text. I don’t see the point of having such a level of detail in a main figure without making it directly understandable within the figure caption. The figure could be simplified, and the additional information could be moved to a supplementary figure.

Great idea. We have improved the caption of Figure 1A to read as follows: “Experimental strains were constructed by inserting GFP, myohemerythrin, or myoglobin at the HO locus in the yeast chromosome. By cutting plasmids in a region of HO homology, the genes are introduced as a high-expression tandem repeat array.” We have also slightly modified Fig 1A to be slightly less busy.

• Minor point: Currently, the figure caption indicates that Fig 1B reports mean with SD. However, I interpreted these to be boxplots reporting IQR, medians, and whiskers (?). Moreover, I couldn’t find the number of biological replicates. Are individual points considered biological replicates? This would need to be clarified.

Good catch. The caption has been corrected to remove an incorrect reference to means and standard deviations. We have clarified that points indicate individual clusters. “Box and whisker plot of oxygen diffusion depth using the mitochondrial MitoLoc reporter in snowflake yeast clusters expressing oxygen-binding proteins or wildtype controls under oxygen limitation. Myohemerythrin and myoglobin expression significantly increased oxygen diffusion depth, but only in the low oxygen environment. Points indicate individual clusters.”

• Minor point: It could be convenient to consider making images accessible to colourblind readers. The reported micrographs will convey little if no message at all. I list the common potential colorblind-friendly two-colour alternatives:
Green/Magenta, Yellow/Blue, and Red/Cyan. Fig 3 has a similar issue but is less limiting than in Fig 1C. I am sure you already know, but in ImageJ, you can recolour individual channels easily (Image>Color>Channel Tools).

This is an excellent point! Having now run these subfigures through a colorblindness simulator, we found that the old images were basically monochrome for those with this condition. We had no idea! Adjusting color for colorblindness improves figure 1C drastically. We have adjusted it as follows, with red fluorescent protein in magenta and green fluorescent protein in cyan, such that colocalization appears clearly white rather than along a spectrum of yellows:

Figure 3, to our eyes, largely escapes problems when run through a colorblindness simulator and has been left as-is, other than making the O_2_ consumption curve of figure 1A dotted to match figure 3B better and differentiate the curves that are most similar under colorblindness simulation. See below:

2B reports the exciting contrast relative to the fitness gains between low and supplemental O2 in normal and small snowflakes. However, I found it difficult to understand why this result was unexpected, even considering the cited references (Lines 156-158).

Good point- you’re not alone, the AE similarly was confused by this. We expanded upon our logic in the overall comments above, and edited the paper to make this more clear. We have expanded on our reasoning in the introduction as follows: “The finding that normal snowflake yeast clusters gained the greatest benefit from oxygen-binding protein expression under supplemental oxygen rather than low oxygen was surprising. We expected the impact of oxygen limitation to be most severe under low oxygen conditions like those that may have suppressed the origins of animals [14, 46, 47]. Why should proteins that are known for alleviating oxygen limitations be more beneficial when oxygen is plentiful?”

Normal heme-expressing snowflake yeast gains a fitness advantage under low O2 levels relative to the wild type (Lines 138-142). Moreover, the significant difference in permeability shown in Figs 1B and C is detected under low O2 in contrast to the supplemental O2 level condition, which shows no significant difference.

You raise an important point about the relationship between our MitoLoc imaging and fitness data. While we see the largest fitness benefits at high oxygen levels, we don't detect significant differences in MitoLoc colocalization under these conditions. This apparent discrepancy likely reflects limitations in our imaging approach rather than a contradiction in the biology. We note that the spread of MitoLoc-indicated diffusion depths is quite large compared to the spread of relative finesses observed in our competition experiments, and the number of observed clusters significantly smaller. The MitoLoc system also requires sufficient proton-motive force to show colocalization, but we don't know precisely how this threshold relates to the oxygen concentrations that supports biologically significant growth. Given these limitations, we trust the detailed findings of competition experiments over those of MitoLoc imaging. Even so, we note that MitoLoc data does recapitulate another aspect of our metabolic models, in that oxygen diffusion depths are significantly higher in supplemental than low oxygen. We have nonetheless addressed this discrepancy briefly in our discussion section: “While MitoLoc imaging showed diffusion enhancement in low-oxygen environments, we found in both competition experiments and biophysical simulations that oxygen-binding proteins provide relatively little benefit to small organisms at low oxygen levels and instead provide the greatest fitness benefit to larger organisms under higher oxygen levels.”

Main-minor point: I consider the O2 levels the primer limiting factor for its diffusion, even if myoH or myoG are abundant. This means that O2 needs to be available to these proteins to observe any effect. In low oxygen conditions, O2 is likely enough only to sustain the physiological activity of cells sitting at the multicellular group periphery.

The results obtained from the model go in this direction (Fig. 3B). The presence/absence of heme proteins is relevant if enough oxygen can be transported. This is also congruent with the observation that, in small snowflakes, there is (yet a non-significant) increase in fitness at low O2 for myoglobin.

Importantly, my point is still congruent with the authors’ conclusion, which I find valid and interesting; I am only raising the question of whether the results from the biophysical model could already be expected by considering the experimental data and previous literature. My specific knowledge of the evolution of multicellularity and the constraints imposed by oxygen availability is likely less than that of the authors; thus, I could be missing some additional information to properly follow the above-mentioned transition.

It is certainly true that oxygen must be present before oxygen-binding proteins can have any effect on fitness. We believe that the conclusions reached in this paper regarding them being more important at high oxygen than low are interesting in the light of their roles in many modern organisms, and in the light of the timing of their origins in the Ediacaran period. We have reframed our discussion of the results as follows:

“Oxygen-binding proteins appear to have originated in the common ancestor of bilaterian animals, a clade characterized by complex anatomy and thick tissues. However, the role of oxygen-binding proteins as a key innovation facilitating the early evolution of large size has remained unclear. Much previous work has suggested that the evolution of multicellular size and complexity was limited by the availability of oxygen[12, 14, 46], and many modern organisms regulate myoglobin levels with increasing expression during hypoxia[62, 63]. This limitation would be consistent with work on the *de novo* evolution of multicellularity in the snowflake yeast model system, in which O_2_ limitation strongly impeded the evolution of larger, tougher multicellular organisms[17, 64]. Facilitated oxygen diffusion, mediated by O_2_-binding proteins, could have lifted this constraint when oxygen was low but present, allowing small multicellular organisms to overcome the constraint of low environmental oxygen.

Our results are not consistent with this hypothesis. While MitoLoc imaging showed diffusion enhancement in low-oxygen environments, we found in both competition experiments and biophysical simulations that oxygen-binding proteins provide relatively little benefit to small organisms at low oxygen levels and instead provide the greatest fitness benefit to larger organisms under higher oxygen levels. This is at first counterintuitive – why would facilitating the diffusion of oxygen not provide the greatest benefit when oxygen is least available? This can be explained by the fact that at high oxygen, the biggest oxygen limitations faced by an simple organism without a circulatory system are *anatomical*, arising from diffusional constraints of abundant oxygen across the organism, rather than *environmental*. Even when there is copious oxygen available in the environment, diffusion through rapidly metabolizing biomass nonetheless depletes it before it can reach deeply into tissues it could theoretically feed and enhanced diffusion is extremely advantageous. In contrast, when environmental oxygen is scarce, there is little metabolic benefit to be gained even by a considerable proportional increase in diffusivity because insufficient oxygen comes into contact with the surface of the organism to theoretically feed deeper tissues.”

Additional minor points:

Fig. 3 – Expressing ‘cluster radius from the centre’ as negative values seems odd (i.e., reporting distances using negative values). Since all graphs are symmetric to 0, they could be halved (as in Fig S3).

Thank you for this thoughtful suggestion about the axis presentation. We chose to maintain the symmetrical presentation around zero in Figures 3A and 3B because we believe it helps readers visualize these graphs as representing a complete slice through a spherical cluster, with negative and positive values showing distance from the center in opposite directions. While your suggestion to show only half the radius (as we did in Figure S3) would be more economical, we felt the complete visualization better helps readers understand the spatial relationships in our three-dimensional system. We have, however, improved the clarity of the axes based on your feedback - we've relabeled the X-axis to read "Position relative to center (µm)" and corrected the Y-axis units for O_2_ consumption rate. Thanks!

Line 157 – Intermediate is likely low in this context.

Great catch, we have corrected the oxygen labeling.

Methods – It could be relevant to cite or describe how distances were measured in Fig. 1B.

We agree, we have cited and clarified how distances were measured as follows: “The diffusion depth of oxygen for each strain was determined as described in Bozdag et al., 2021[17]. Clusters were flattened under a coverslip before imaging. Flattened clusters were segmented automatically in imageJ, and inner anaerobic regions not exhibiting red and green colocalization were manually segmented within them. Areas were measured, and a summary statistic of oxygen diffusion depth was calculated by subtracting the radius of a circle with the area of the anaerobic region from the radius of a circle with the area of the full cluster.”

Thank you for your deeply thoughtful and constructive review. Your insights helped us clarify both our methods and our interpretations, making the work more rigorous and accessible to readers.

Reviewer #2:

Wong et al. report experiments suggesting that oxygen-binding proteins only became evolutionary advantageous once oxygen was an abundant resource, and in multicellular organisms that were complex enough for diffusion of oxygen alone to be insufficient as a delivery mechanism to inner cells. The authors have backed up the experimental work with mathematical modelling. Wong et al. state that the results challenge some of the current thinking on the topic, which, according to the authors, is based on intuitive notions.

I find the data and discussion in alignment with the scope of Short Reports at PLOS Biology. The data is compelling and clearly presented, and it supports the conclusions well. So, I don't have any major criticism.

I should disclose that I'm unable to evaluate the methodology regarding mathematical modelling.

My commentary below is aimed at enhancing clarity.

We greatly appreciate your positive assessment of our work and your thoughtful suggestions for improving its clarity. We have carefully addressed each of your suggestions throughout the manuscript, from improving precision in our discussion of the Proterozoic period to enhancing our methods descriptions and figure presentations. The manuscript is now clearer and more accessible while maintaining its scientific rigor.

These are presented in sequential order:

Line 26-30. Boring billion. I wish to encourage you to be a little more precise in these statements. A lot of evolutionary important events occurred within this period, including the origin of eukaryotes and their early diversification into many lineages. It sounds as if the world was only inhabited by prokaryotes and algae from 2.5 to 0.5 billion years ago.

Line 27. Boring billion refers more specifically to the range between 1.8 to 0.8 billion years ago, not the entire Proterozoic. It should be noted that the Proterozoic is actually two billions! :)

I wonder if you can replace some of the references 1 to 7 with something more recent that captures better the state of knowledge. Have a look at this reference, just one example: <https://www.nature.com/articles/s41586-023-06170-w.\>

We appreciate this helpful comment about the rich evolutionary history of the Proterozoic. We have removed the "boring billion" terminology and now refer specifically to the Proterozoic period. While remarkable discoveries continue to illuminate early eukaryotic evolution during this time, our study focuses specifically on the origins of complex multicellularity. We have therefore focused our citations on clear multicellular algal fossils and potential early animal remains, including microfossils that may represent relatives of metazoans or early sponges, as these are most relevant to our work on oxygen utilization in multicellular organisms.

Line 32. Reference [8] is not displaying correctly in the reference list. Also, what do you mean with "linked"? It could be read as a causal relationship. Try to use more precise language if possible.

Good catch! The references have been repaired. We agree with the note on language, and now state that “It has long been hypothesized that the dramatic rise in atmospheric oxygen levels marking the end of the Proterozoic, from ~1% of modern atmospheric levels to near-modern concentrations, was necessary for or triggered the evolution of animal multicellularity[12-14].”

Line 73-74. It might be a good idea to explain why this is "unexpected". For example, has the opposite been suggested by others previously? If so, can you provide some reference for this? Or was your original hypothesis the opposite to what you found when you set out to do these experiments?

Thank you for this thoughtful point about clarifying our initial expectations. Based on our previous work (Bozdag et al., 2021) showing that low oxygen strongly constrains the evolution of larger multicellular clusters, we initially hypothesized that oxygen-binding proteins would provide their greatest benefit under low-oxygen conditions. Interestingly, while there is extensive literature on oxygen-binding protein function across different oxygen concentrations, and their presence in organisms adapted to hypoxic conditions (e.g., Mills & Canfield 2014, Sperling et al. 2015), we do not know of any papers in that have predicted or tested the relative fitness benefits of these proteins under different oxygen regimes. We have now clarified our reasoning in the manuscript.

In the Introduction:

“We expected that while enhanced oxygen diffusion would benefit snowflake yeast at all oxygen levels, the benefit would be largest at low oxygen because this environment presents the harshest limitation to the evolution of increased size in the snowflake model system[17]. However, as we examine the fitness consequences of facilitated diffusion, we find an unexpected result: oxygen-binding proteins confer the greatest advantage for large multicellular clusters not when oxygen is rare and diffusion is slow, but when it is abundant.”

In the Discussion:

“Oxygen-binding proteins appear to have originated in the common ancestor of bilaterian animals, a clade characterized by complex anatomy and thick tissues. However, the role of oxygen-binding proteins as a key innovation facilitating the early evolution of large size has remained unclear. Much previous work has suggested that the evolution of multicellular size and complexity was limited by the availability of oxygen[12, 14, 46], and many modern organisms regulate myoglobin levels with increasing expression during hypoxia[62, 63]. This limitation would be consistent with work on the *de novo* evolution of multicellularity in the snowflake yeast model system, in which O_2_ limitation strongly impeded the evolution of larger, tougher multicellular organisms[17, 64]. Facilitated oxygen diffusion, mediated by O_2_-binding proteins, could have lifted this constraint when oxygen was low but present, allowing small multicellular organisms to overcome the constraint of low environmental oxygen.

Our results are not consistent with this hypothesis. While MitoLoc imaging showed diffusion enhancement in low-oxygen environments, we found in both competition experiments and biophysical simulations that oxygen-binding proteins provide relatively little benefit to small organisms at low oxygen levels and instead provide the greatest fitness benefit to larger organisms under higher oxygen levels. This is at first counterintuitive – why would facilitating the diffusion of oxygen not provide the greatest benefit when oxygen is least available? This can be explained by the fact that at high oxygen, the biggest oxygen limitations faced by an simple organism without a circulatory system are *anatomical*, arising from diffusional constraints of abundant oxygen across the organism, rather than *environmental*. Even when there is copious oxygen available in the environment, diffusion through rapidly metabolizing biomass nonetheless depletes it before it can reach deeply into tissues it could theoretically feed and enhanced diffusion is extremely advantageous. In contrast, when environmental oxygen is scarce, there is little metabolic benefit to be gained even by a considerable proportional increase in diffusivity because insufficient oxygen comes into contact with the surface of the organism to theoretically feed deeper tissues.”

This revision helps readers understand both our initial reasoning and why our results were surprising. Thanks!

Consider your introduction in light of your discussion section. You might want to explicitly state at the beginning what had been hypothesized before, or what the expected/intuitive hypothesis was. Then, explain how your work disproves it.

Thank you for the suggestion for clarity in our introduction. To our knowledge, the relationship between the origins of oxygen-binding proteins and the environment of multicellular animals is not well explored in the literature. We hypothesized from first principles that they would be most useful under low oxygen conditions due to previous work from Bozdag et al., 2021 showing that multicellular size was most strongly limited in the snowflake yeast model system in low-oxygen conditions. We sought to test this hypothesis, and disproved it, revealing an unexpected dynamic between the evolution of oxygen diffusion and multicellular size. This is reflected in the modified introduction quoted above.

Lines 94-95. I'm not sure how broadly used this method is but consider rephrasing this for extra clarity. For example, say that, according to the reference, COX4 is imported into mitochondria proportional to the membrane potential, which is proportional to the rates of respiration (if I understood correctly). It just feels a bit vague.

We agree that a more precise description of the MitoLoc system's mechanism would be valuable to readers. We have revised this section to explicitly explain how the system works, clarifying that:

“The MitoLoc system uses a constitutive GFP tag on the outer mitochondrial membrane protein TOM70 to visualize mitochondrial morphology, and an mCherry tag on the inner membrane COX4 protein. This protein’s rate of import from the cytoplasm to the inner membrane is dependent on mitochondrial inner membrane potential, allowing colocalization of these two tagged proteins to detect membrane potential indicative of high respiration rates[43].”

While our original methods were accurate, the revised description provides a clearer mechanistic explanation of how the system indicates respiratory activity through the relationship between membrane potential, protein import, and fluorescent protein colocalization.

Line 98-100. I wonder if you can use more precise language here. For example, "after 7 hours the concentration of oxygen in the culture was below 5% saturation..." It would be ideal if you could mention the concentrations of dissolved oxygen in concentration units, rather than as a percentage of saturation.

We agree completely that providing oxygen levels in molar concentrations alongside percent saturation would make our results more comparable across studies that use different conventions. We have calculated the corresponding molar concentrations based on oxygen solubility in water at 30°C, where 100% saturation is close to 0.25 mM, the figure we used for saturation in our diffusion models. This allows us to report both relative and absolute measures throughout the manuscript, making the experimental conditions more concrete and directly relatable to our modeling work.

Line 274. "Linked", how? In which way were these linked?

Good point. We have clarified our frequent use of the word ‘linked’. We now state that “Much previous work has suggested that the evolution of multicellular size and complexity was limited by the availability of oxygen”

Line 275, "This has been…" it's not clear what "this" refers to.

Nice catch. We have clarified, stating that “This limitation would be consistent with…”

Lines 271-280. I just get a feeling that this works better in the introduction*.*

We agree that this point could be better made in the introduction ahead of time. We have expanded a discussion of the apparent history of oxygen-binding proteins in the introduction (see our response to your comment on lines 73-74 for this text).

Line 303. "In response to", not precise enough. Perhaps evolved "as an adaptation to"...

Good point. We have clarified as follows: “We therefore suggest an alternative hypothesis for the role and timing of the origins of oxygen-binding proteins in the Metazoa: that they may have evolved not as an adaptation to circumvent low atmospheric oxygen early in animal evolution, but instead became favored to emerge due to rising oxygen levels during the Neoproterozoic, allowing multicellular organisms to better exploit the increased metabolic potential of abundant environmental oxygen”

Lines 330-332. It isn't clear the rationale for choosing myohemerythrin and myoglobin from the mentioned organisms. Can you provide a brief statement on this? Are these standards in the field?

This is an excellent point, as understanding why specific model proteins were selected helps readers evaluate our experimental approach. We have added context about our protein selection in the introduction, where we now state:

“We heterologously express the well-studied model oxygen-binding proteins myoglobin from sperm whales (*Physeter macrocephalus*)[37] and myohemerythrin from peanut worms (*Themiste zostericola*)[38] in oxygen-limited snowflake yeast (modified *Saccharomyces cerevisiae*), a model system of diffusion-limited multicellularity capable of rapid *in vitro* evolution[39-42].”

These specific proteins were chosen because they represent the most thoroughly characterized examples of their respective classes. Sperm whale myoglobin became the standard model for myoglobin studies historically because whale muscle contains exceptionally high concentrations of the protein, making it easier to purify in the era before heterologous expression. Similarly, peanut worm myohemerythrin, while not as extensively studied as myoglobin, is the most well-characterized member of the hemerythrin family. Using these well-studied proteins allows us to draw upon decades of biochemical and biophysical characterization in interpreting our results.

Line 351. Please rephrase for clarity. "Previously described" Where? Could you be more precise?

This plasmid was cut with AfeI or BsaAI, as previously described for GFP, myoglobin, and myohemerythrin expression. It now reads: “This plasmid was cut for genomic insertion at the *HO* locus with AfeI or BsaAI to generate two heterozygous MitoLoc and oxygen-binding protein expressing snowflake strains (see strain list).”

Line 398. What did you use to bubble air?

Our air supply originated from the laboratory-building-supplied pressurized air spigots, which was humidified by bubbling through sterilized water before sparging into the bottom of culture tubes via 14-gauge needles, substantially as described in Miller et al., 2013. This has been clarified as follows:

“10 mL cultures were grown in a low oxygen environment shaking at 250 RPM at 30°C, and supplemental oxygen cultures were aerated by bubbling humidified laboratory air through the growth media at 30°C using 14-gauge needles as described in Bozdag et al., 2021[17] with protocols for the use of multiplexed chemostat arrays[44].”

Line 400-401. It isn't clear how you do this. Use more precise language. How do you determine depth from an area measurement, when these cluster of cells are highly irregular? Might be a good idea to provide a visualization.

We agree. We measured the depth of aerobic respiration as described in Bozdag et al., 2021. We have clarified the method as follows: “The diffusion depth of oxygen for each strain was determined as described in Bozdag et al., 2021[17]. Clusters were flattened under a coverslip before imaging. Flattened clusters were segmented automatically in imageJ, and inner anaerobic regions not exhibiting red and green colocalization were manually segmented within them. Areas were measured, and a summary statistic of oxygen diffusion depth was calculated by subtracting the radius of a circle with the area of the anaerobic region from the radius of a circle with the area of the full cluster.”

Line 404-408. Is the number of cells measured by OD? Do you expect the number of cells to be approximately equal per unit of volume? Why don't you just simply say the exact volumes of culture grown at the start of the experiment, and the volumes used for each step of the experiment?

You raise several excellent points about the need for precise methodological details that would enable others to replicate our work. We have substantially expanded our methods section to include all the specific details about culture volumes and growth conditions.

All experimental strains were first grown to stationary phase in appropriate media for 24 hours, followed by a second day of growth after reinoculation. While we did not measure exact cell densities by OD, growing to stationary phase helped ensure consistent cell concentrations across strains. On experiment day 0, we combined equal volumes of experimental and GFP control strains, with initial ratios consistently falling within 5-10% of 1:1 as measured by our imaging analysis. We then inoculated:

- 100 μL of culture mixture into 10 mL fresh media for multicellular competition experiments
- 50 μL of culture mixture into 10 mL fresh media for unicellular and β-estradiol induction experiments
- These volumes were maintained for daily passages over the three-day competition period

Importantly, while there was minor variation in initial ratios, our fitness calculations are based on the change in population ratios between day 0 and day 3, making them robust to any variation in initial genotype frequency, as fitness is normalized by actual measured changes in frequency between day 0 and 3. Thanks for suggesting we clarify this! We include an updated methods section below your next comment.

Line 411/Line 414-415. How did you measure the final proportions for each population? How many cells do you count? Do you count with a software or manually? How many replicates?

Thes are all extremely important things to clarify in our methods. Five replicate populations were inoculated from each initial starting strain mixture, which has been clarified. Regarding this and your previous methodological questions, this part of the Methods section now reads:

“All strains were competed against GFP-bearing control lines with the same *ACE2*, *BUD8*, and *HEM3* genotypes. All yeast were grown in 10 mL of appropriate culture media (YEP-Glycerol or YEP-Dextrose) at 30°C shaken at 250 RPM. Low oxygen cultures were stirred in a shaking incubator without aeration, or aerated as described above from Bozdag et al., 2021 with protocols for the use of multiplexed chemostat arrays[44]. Monocultures of all necessary strains were grown for 24 hours to stationary phase in YEP-Glycerol or YEP-Dextrose as needed for one day, and then 100 microliters reinoculated into another tube for a second day before experiments began. On experiment day 0, equal volumes of GFP-expressing control yeast and experimental yeast were mixed to form the starting population. Five replicate populations were inoculated from each mixture. A total of 100 microliters of this mixture was inoculated into 10 mL of fresh media at the start of each competition in the case of multicellular constitutive expression experiments, while 50 microliters was inoculated for unicellular competitions and β-estradiol induction competitions. The populations were passaged for a total of three days, inoculating the same volume of culture into fresh media every twenty-four hours. Upon initial mixing of monocultures on day 0, and after day 3 of growth, 25 µl of multicellular cultures were diluted into 1 mL of water on 12-well plates and allowed to settle. Tiled bright-field and FITC channel images were obtained via the Nikon Eclipse Ti inverted microscope, until 1000-3000 clusters were imaged. Clusters were automatically segmented via ImageJ scripts, segmentation errors manually corrected, and clusters under 250 square microns automatically excluded. Each cluster was automatically classified as GFP or non-GFP based on fluorescence level using an R script, thresholded based on the FITC channel intensity with minimal cluster density. Unicellular cultures were instead counted via flow cytometry and analyzed via FloMax 3.0 (Partec, Göttingen, Germany).”

Line 412. "finding the ratio of Malthusian parameters" you provide three references for this. Can you be more precise as to where exactly the complete information needed can be found? Think about a new student wanting to reproduce these experiments and measurements.

Good call. We have simplified the citation to a single reference (Lenski et al., 1991) as it contains the precise equation used. We have also written out the equation we used in our paper so readers do not have to go searching for it:

“After measuring the final proportions of GFP and non-GFP clusters or unicells for each population, we calculated the relative fitness of each strain relative to its GFP control by finding the ratio of their Malthusian parameters [68]. Specifically, given an initial population fraction of *p_0_*, a population fraction on passage *n* of *p_n_*, and a dilution factor of *f_d_*, the final relative fitness *W* of that population fraction is expressed as:

$W=\frac{ln({f_{d}}^{n}\cdot p_{n}/p_{0})}{ln({f_{d}}^{n}\cdot(1-p_{n})/(1-p_{0}))}$”

Line 669. "vanishingly" can you be more precise? I would personally equate "vanishingly low" with few parts per million.

We agree this language is imprecise. We have clarified the oxygen levels, both in terms of percent saturation and in terms of millimolar concentrations as used elsewhere in our manuscript. 0.25 mM is close to the solubility of oxygen in water at 30 degrees Celsius, and corresponds to approximately 4 ppm or 4 mg/L.

Lines 671-672. Fig. S1 Do you mean that this data is taking from ref. 11? It isn't clear. Please clarify this in the materials and methods. Also what oxygen-detection method was used to produce the traces in Fig. S1.

The data was taken from our original reference 11, current reference 17, Bozdag et al., 2021, which has been clarified. Each trace is the average of five replicate oxygen measurement traces from batch culture of yeast. Oxygen levels were measured with fiber-optic optodes (FireString O_2_, PyroScience, GmbH, Germany) as described in the text of Bozdag et al., 2011. Together with the above clarification, the supplemental caption now reads:

“Without supplemental oxygen the majority of a growth cycle is spent at oxygen levels under <5% saturation (<0.0125 mM). With supplemental oxygen, the majority of a growth cycle is spent at greater than 50% oxygen saturation ( >0.125 mM) and average oxygen levels do not drop below 32% saturation (0.08 mM). Data taken from Bozdag et al., 2021[17]. Five replicate measurements of oxygen profiles taken with fiber-optic optodes were averaged to produce each oxygen profile.”

Thank you for your careful and constructive review, particularly your suggestions for improving precision in our language and clarity in our methods. Our paper is much improved as a result!

Reviewer #3:

In this work, the authors use the experimentally evolved snowflake yeast to assess the importance of oxygen-binding proteins in bypassing the diffusion barrier within a multicellular colony. This is an interesting and appealing system, be it a genetically tractable system that can be manipulated in various ways to ask simple question about the transition to multicellularity. In that sense, the experiment done here is elegantly thought and snowflakes are very suitable to tackle this question. The unintuitive result obtained, in which bigger-sized colonies have a greater fitness benefit at high O2 compared to small cells is somewhat supported by the data, and the use of a computational model seems to support these results. Overall, it's an interesting and exciting result, and would be suitable for a short report in PloS Biology. However, some concerns below need to be addressed to strengthen some conclusions and make this a better study.

Thank you for your positive evaluation, and suggestions for improvement. As detailed below, we have conducted new experiments examining protein expression costs and growth dynamics that validate our key conclusions while revealing important limitations of our approach (i.e., you crank up globin expression too much and the yeast aren’t happy).

Growth dynamics of budding yeast cells with plasmids with myoglobin and myohemerythrin. Many experiments assume that these strains grow at a comparable rate as the WT, although there is no data for this. I think ensuring that cells grow at same speed is critical for measurements of O2 depth but also fitness. I would recommend assessing the growth dynamics in the snowflakes Ace2- as well as in the single-cell ancestor Ace2+. Ensuring that these strains grow at similar speed is critical for interpreting following data.

Thank you for this excellent suggestion. You are absolutely right that baseline growth differences could confound interpretation of both our oxygen diffusion and fitness measurements. We have now performed extensive controls comparing growth of unicellular strains expressing myoglobin, myohemerythrin, or no oxygen-binding protein against GFP-expressing controls.

First, we competed these strains in glucose media (YEP-Dextrose) where aerobic capacity is unimportant, allowing us to isolate the basic costs of protein expression. As seen in Figure S3, myohemerythrin expression showed a slight 0.7% fitness advantage over GFP controls, while myoglobin fitness was equivalent to GFP, suggesting minimal protein synthesis costs.

We then tested these strains in YEP-Glycerol under both low and supplemental oxygen conditions, mirroring our experiments with multicellular clusters. Under low oxygen, unicellular strains showed similar fitness effects to small snowflake clusters. However, under high oxygen, myoglobin-expressing unicells showed a slight fitness deficit, possibly due to competition for heme between myoglobin and respiratory chain components. Despite this baseline cost in unicells, our key findings remain robust: oxygen-binding proteins provide their greatest fitness benefit in large multicellular clusters under high oxygen conditions, while showing neutral or negative effects in unicells and small clusters. See our new Figure 2B:

Thanks again for this great suggestion, we think it really strengthened our paper.

The measurements of oxygen diffusion depth in principle should be alright done using microscopy. However, to strengthen this I recommend a better explanation of the measurement's method, maybe with a drawing and comparative kymographs. Another point that I think is critical, is accounting for overall colony size. Small size differences between colonies may allow for increased variability. Maybe measuring depth per colony and normalizing it per colony diameter for each colony would better help to obtain a more robust measurement.

We agree. We have expanded our explanation of the measurement method, and cited Bozdag et al., 2021 for the method. It now reads as follows: “The diffusion depth of oxygen for each strain was determined as described in Bozdag et al., 2021[17]. Clusters were flattened under a coverslip before imaging. Flattened clusters were segmented automatically in imageJ, and inner anaerobic regions not exhibiting red and green colocalization were manually segmented within them. Areas were measured, and a summary statistic of oxygen diffusion depth was calculated by subtracting the radius of a circle with the area of the anaerobic region from the radius of a circle with the area of the full cluster.”

The small snowflake colonies are generated through a bud8 mutation. Although there is no known link between bud8 and metabolism, we can also imagine that for some indirect reason, bud8 interacts negatively with myoglobin etc. It would strengthen the conclusions if there was another mutation, maybe *wee1* or *for3*

You raise a valid concern about potential unknown interactions between *bud8* and our oxygen-binding proteins. Rather than construct additional small-cluster mutants, we addressed this concern by examining protein expression effects in unicellular strains. These experiments serve as an even more stringent test than alternative small-cluster mutants, as unicells completely eliminate any confounding effects of multicellular organization while still allowing us to test baseline costs and benefits of oxygen-binding protein expression. As detailed above, these unicellular controls support our key finding that the benefits of oxygen-binding proteins emerge specifically in large multicellular clusters.

The predictions from the computational model concerning the linear increase with increase in amounts of myoglobin should be easily testable experimentally. Although I understand this is a short report, it could be envisaged to test this using inducible promoters or promoters of different strengths.

Thank you for suggesting this important test of our model predictions. We have taken two approaches to address this: First, we have revised our model interpretation to more accurately reflect the predicted relationship between myoglobin levels and fitness, now emphasizing rapidly diminishing returns at low oxygen levels and slowly diminishing returns at high oxygen levels. Second, we tested these predictions experimentally using an inducible promoter system to vary myoglobin expression (Figure 4). While we observed a fitness benefit at moderate expression levels under high oxygen conditions, extremely high expression became detrimental, likely due to competition between myoglobin and respiratory proteins for limited cellular heme. This reveals an important limitation of our experimental system - achieving the predicted linear benefits of increased myoglobin expression may require concurrent optimization of cellular iron and heme metabolism.

*Small things:
- mutant names should be italic
- Line 15 to 8 is copy pasted from abstract, a variation is better for reader.
- You need a ref for Line 27th
- Statistical test description can be added to figure legends rather than within Figure callouts. It breaks the reader flow
- Fig S2: the scale bars are not of similar sizes.*

Thank you for these helpful formatting suggestions. We have:

- Italicized all mutant and gene names throughout the manuscript
- Added citations from Dos Reis et al. (2015) and Buatois et al. (2016) to support Line 27
- Moved statistical test descriptions to figure legends to improve readability
- Regenerated Figure S2 with internally consistent scale bars (see below)
- Regarding Lines 15-8: these appear within the abstract itself, so the repeated text is appropriate in this context.

Thank you for your thoughtful and thorough review. Your suggestions led to a number of important improvements in our manuscript, particularly the addition of unicellular controls that helped validate our key findings while revealing important limitations of our experimental system. The combination of your technical suggestions and broader conceptual feedback has helped us create a more rigorous and complete story. We really appreciate the time and care you took in reviewing our work.
